# Screening for putative essential genes in *Brucella abortus* strains A19 and S2308 using transposon mutagenesis

**DOI:** 10.52601/bpr.2025.250050

**Published:** 2026-10-31

**Authors:** Siwei Guo, Weidong Bu, Fangfang Guo, Dianyu Li, Liyuan Liu, Xiaxia Liu, Yuefeng Chu, Jian Xu

**Affiliations:** 1 State Key Laboratory for Animal Disease Control and Prevention, College of Veterinary Medicine, Lanzhou University, Lanzhou Veterinary Research Institute, Chinese Academy of Agricultural Sciences, Lanzhou 730046, China; 2 Institute of Animal Husbandry and Veterinary Medicine, Beijing Academy of Agriculture and Forestry Sciences, Beijing 100097, China

**Keywords:** Brucella, Transposon mutant library, Transposon sequencing (Tn-seq), Essential genes

## Abstract

*Brucella* is a zoonotic pathogen that causes substantial economic losses in the livestock industry. However, the categorization of functional genes in *Brucella* requires further refinement. This knowledge gap was addressed by classifying gene essentiality through constructing a transposon mutant library. Mariner transposon mutant libraries were independently constructed for *Brucella abortus* A19 and S2308. Transposon sequencing was used to visualize the insertion sites within the mutant library, which revealed a uniform transposon distribution in both genomes and high library saturation. Furthermore, 451 and 393 putative essential genes were identified for *B. abortus* A19 and S2308, respectively. The functional enrichment analysis of these genes revealed that cofactor biosynthesis was the most highly represented, followed by ribosomal biogenesis, carbon metabolism, amino acid biosynthesis, and oxidative phosphorylation. Many of these genes encode known antimicrobial drug targets. This study provides a foundational resource for *Brucella* research, and the mutant library serves as a tool for screening functional *Brucella* genes.

## INTRODUCTION

*Brucella* belongs to the phylum *Proteobacteria* and is a facultative intracellular parasite with a Gram-negative coccobacillus morphology (Corbel 1997). Brucella lacks endospores, capsules, and flagella. Brucellosis is caused by *Brucella* and is thus one of the most important zoonoses worldwide (Hasanjani Roushan and Ebrahimpour 2015). Brucellosis causes abortion and fever, posing serious threats to human and animal health. *Brucella* is classified into six species and 19 biovars based on pathogenicity and biochemical characteristics, among others (Abdallah *et al*. 2003). *Brucella melitensis*, *Brucella abortus*, and *Brucella suis* are the most prevalent and pathogenic (Alakavuklar *et al*. 2023). *B. abortus* A19 (vaccine strain) and S2308 (virulent wild-type strain) were selected for comparative analysis.

The persistent global burden of brucellosis underscores critical gaps in our understanding of *Brucella* biology. Decades of research have yet to fully elucidate the genetic requirements underlying bacterial survival and pathogenesis — knowledge essential for developing novel therapeutic interventions. Essential genes, defined as those indispensables for growth and survival under specific conditions, represent particularly attractive drug targets as their disruption proves lethal to the pathogen. Systematic identification of these genes therefore provides dual benefits: advancing fundamental understanding of bacterial physiology while revealing vulnerabilities for therapeutic exploitation.

Essential genes are genes that are crucial for the growth and survival of organisms under specific environmental conditions. The methods of identifying essential genes include single-gene knockout, RNA interference, CRISPR/Cas9, and transposon mutagenesis (Hayes 2003). In this study, we constructed a transposon mutant library for screening in *B. abortus* strains A19 and S2308.

A transposon is a discrete DNA segment capable of expressing its transposase. A transposon enzyme recognizes the terminal inverted repeats (TIRs) flanking the transposon, mediates transposon excision, and facilitates transposon transposition within the genome (Kleckner *et al*. 1975). Transposons can be transferred from a donor to a recipient genome via insertion between specific base pairs and are heritable genetic elements (Craig 1997). Transposons have been progressively used for screening the genes of prokaryotic and eukaryotic organisms since the initial discovery of transposons in the maize genome in the 1940s and the construction of the synthetic Tc1/Mariner-type transposon Sleeping Beauty in the late 20th century (van Opijnen and Camilli 2013).

Different transposon types exhibit distinct insertion site preferences: the Mariner transposon randomly inserts into thymine (T)–adenine (A) dinucleotide sites (Cain *et al*. 2020). Mu and Tn5 transposons exhibit a preference for inserting into DNA sequences containing guanine (G) and cytosine (C). The Tn7 insertion pattern is more random, showing a slight preference for A- and T-rich sequences (Green *et al*. 2012). The PiggyBac transposon is active in mammalian cells and randomly inserts at TTAA tetranucleotide junctions (Ding *et al*. 2005).

Highly random transposons are typically selected in molecular biology experiments for constructing highly saturated mutant libraries, ensuring the reliability of the subsequent analyses (Gray *et al*. 2015). Tn5 or Mariner transposons are commonly used for constructing bacterial transposon mutant libraries (Guo *et al*. 2013). Antibiotics are selected, and each bacterium within the mutant library typically harbors only a single transposon insertion site, enabling tens of thousands of mutant clones to be generated. We used the Mariner transposon to construct a saturated mutant library of *B. abortus* A19 and S2308.

Transposon insertion sites must be precisely mapped after constructing a random transposon mutant library. Transposon insertion sequencing (TIS) was developed for this task. TIS is a methodology that combines all transposon insertion mutations with high-throughput sequencing to assess the role and essentiality of each gene within the bacterial genome (Barquist *et al*. 2013). TIS enables the direct sequencing of transposon-flanking regions, simultaneously allowing individual insertion sites to be precisely identified and genomic regions devoid of insertions to be identified. Most or all nonessential genes are expected to harbor transposon insertions in a saturated mutant library; genes lacking insertions are highly likely to be essential. The library can be subjected to selective growth conditions, such as nutrient limitation; the library can be sequenced before and after selective pressure to determine the relative abundance of each mutant. This process facilitates the analysis of gene–phenotype associations under specific conditions (Chao *et al*. 2016).

Transposon sequencing (Tn-seq) is a specific implementation of TIS (van Opijnen and Camilli 2013). Tn-seq is commonly used for sequencing bacterial mutant libraries constructed using Mariner or Tn5 transposons. The type IIS restriction enzyme Mmel was originally used in Tn-seq to digest extracted genomic DNA from the mutant library (Morgan *et al*. 2009). However, this approach generated heterogeneous fragment sizes, some of which could be lost during the subsequent purification or PCR steps, biasing the sequencing results. The current commercial Tn-seq protocols typically involve the use of random fragmentation to shear genomic DNA into 200–500 bp fragments, mitigating the impact of size heterogeneity (Friedrich *et al*. 2017). The Tn-seq leverages massively parallel sequencing, providing highly reproducible and sensitive results. Tn-seq approach allows the contributions of gene fitness contributions in specific environments to be identified without relying on preconstructed knockout arrays (van Opijnen *et al*. 2009).

We employed a Mariner transposon system delivered via bacterial conjugation to construct mutant libraries of *B. abortus* strains A19 and S2308. The subsequent Tn-seq analysis enabled the genome-wide classification of gene essentiality and the preliminary characterization of essential genes.

## RESULTS

### Construction and sequencing analysis of mutant library

The results of three independent conjugation experiments showed that comprehensive transposon insertion libraries were successfully constructed, yielding 84,000 and 64,000 random mutant colonies of *B. abortus* strains A19 and S2308, respectively. Representative aliquots from each library were subjected to high-throughput sequencing analysis following standard quality control procedures. Sequencing generated 17,801,501 and 16,098,379 high-quality reads for the *B. abortus* A19 and S2308 libraries, respectively, with Q30 scores of 85.2% and 85.0%, respectively. This result confirmed that both libraries met the established quality standards for downstream analysis.

The *B. abortus* A19 genome contains 59,925 TA dinucleotide sites within the annotated open reading frames that are potential transposon insertion targets. The results of comprehensive sequencing analysis identified successful transposon insertions at 41,975 TA sites, covering 70.04% of the insertions across the genome. This high insertion frequency demonstrated that the mutant library was nearly saturated, providing robust coverage for determining essentiality (Xiao *et al*. 2023). Similarly, the *B. abortus* S2308 genome harbored 59,789 TA dinucleotide sites within open reading frames, with 37,444 confirmed insertion events detected through sequencing analysis, confirming that this library was also adequately saturated for reliably assessing gene essentiality.

The results of the statistical analysis of the TA site insertion frequencies revealed distinct bimodal distribution characteristics in the saturated transposon libraries. Genes with low and high insertion rates in the *B. abortus* A19 library formed well-separated, nonoverlapping populations at opposite extremes of the distribution spectrum. The saturation characteristics of the *B. abortus* S2308 library were broadly similar to those of the A19 library, albeit at a somewhat lower level. This difference is likely attributable to the smaller size of the S2308 library (Fig. 1). The genes with consistently low insertion frequencies (<20%) were predominantly essential for bacterial viability, whereas genes tolerating high insertion rates (>80%) were classified as nonessential for growth under standard laboratory conditions (Yang *et al*. 2017).

**Figure 1:**
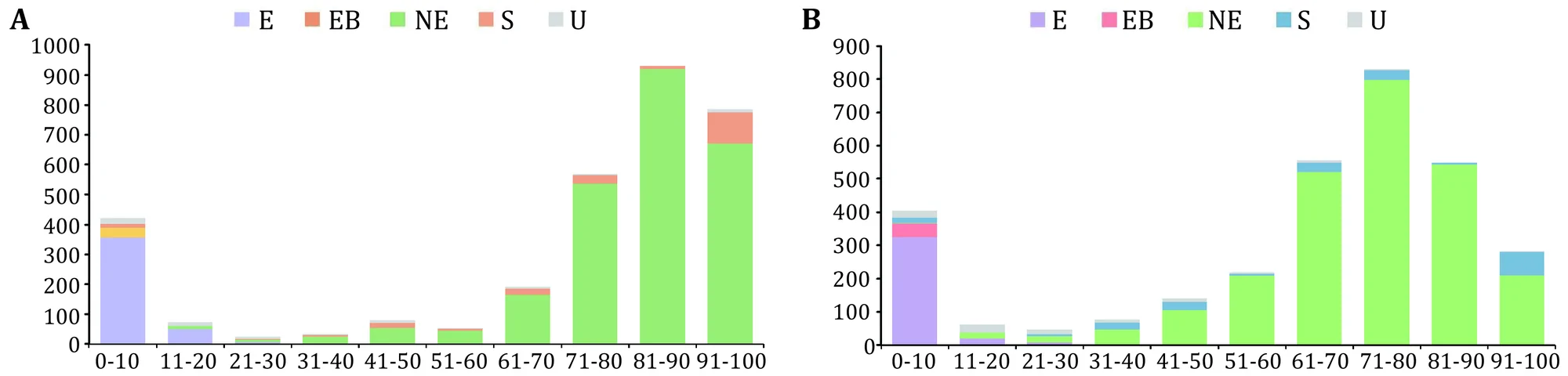
Histogram showing the percentage of TA loci disrupted per gene in the *B. abortus* strain A19 (**A**) and S2308 (**B**) transposon insertion library. The *x*-axis shows the percentage of TA sites per gene that contain a transposon insertion in the *B. abortus* mutant library. The *y*-axis shows the number of genes corresponding to each proportion bin. E: essential based on Gumbel; EB: essential based on binomial; NE: non-essential; S: too short; U: uncertain

The insertion site data were comprehensively visualized using TRANSIT software (version 3.0.1), generating circular genome maps that simultaneously displayed read counts per insertion site, essential gene annotations, nonessential gene classifications, and genes of undetermined essentiality (Fig. 2). These results demonstrated the absence of insertion bias across nonessential genomic regions and indicated the markedly reduced or absent insertion densities within essential gene loci, confirming the reliability of the gene essentiality classifications.

**Figure 2:**
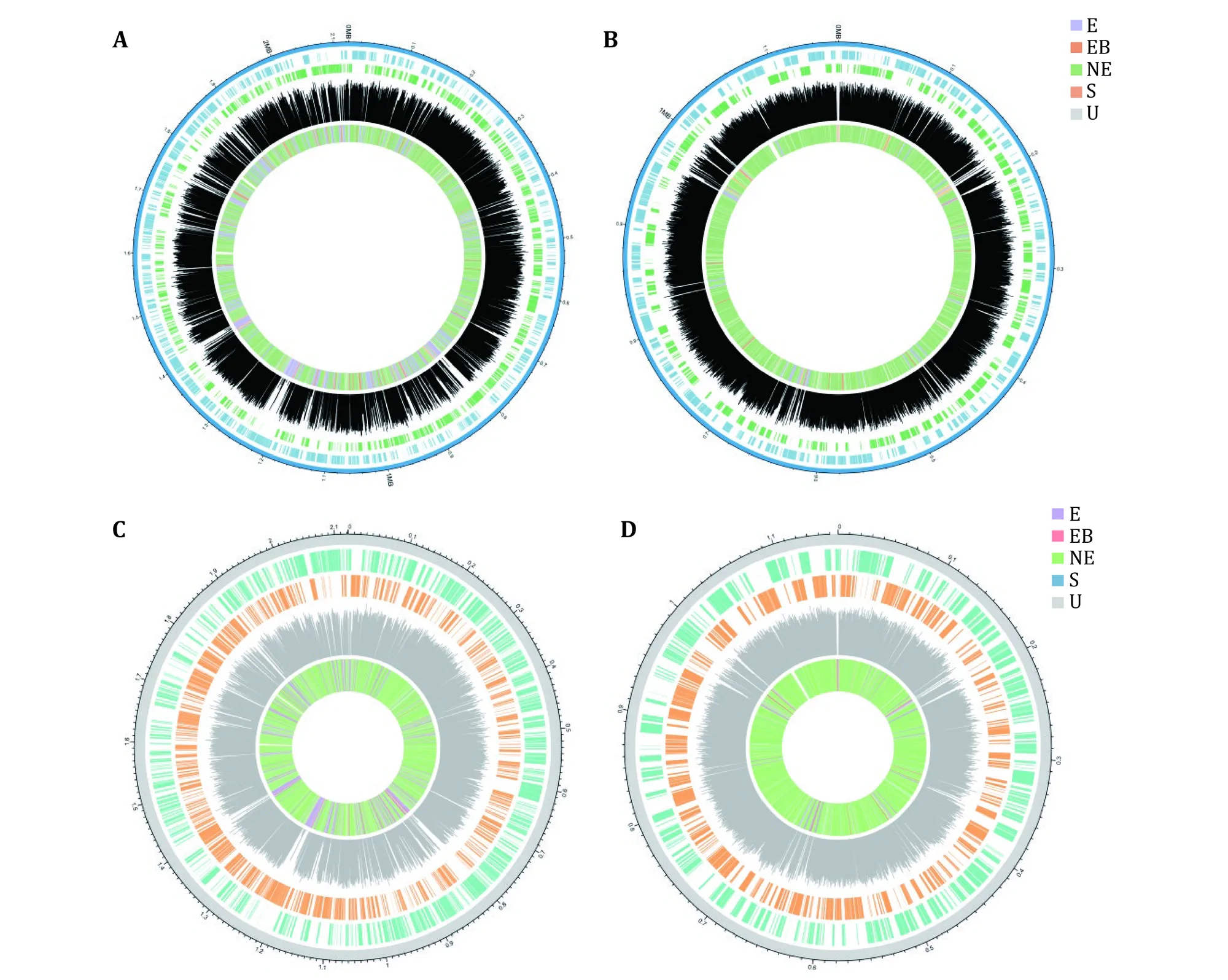
Circular maps of all transposon insertion sites in *B. abortus* genomes of strains A19 and S2308. The outer to inner circles are scale marks of the genome (Mb), coding sequences on the forward strand, coding sequences on the reverse strand, and number of inserted reads per locus from the input, expressed in lg10. The colors in the status labels represent the types of genomic features in the innermost circle of the circular map. E: essential based on Gumbel; EB: essential based on binomial; NE: non-essential; S: too short; U: uncertain. **A**,**B** Transposon insertion sites on *B. abortus* A19 chromosomes. **C**,**D** Transposon insertion sites on *B. abortus* S2308 chromosomes

The genes were quantitatively categorized using Gumbel statistical analysis, identifying 451, 2428, 82, and 211 essential genes, nonessential genes, genes of ambiguous essentiality, and genes with insufficient sequences for reliable analysis, respectively, in *B. abortus* A19. This analysis of *B. abortus* S2308 revealed 393 essential genes, 2466 nonessential genes, 98 ambiguous genes, and 212 genes with inadequate sequence lengths for classification (Fig. 3). The distribution of the essential genes across the bipartite *Brucella* genome showed pronounced chromosomal bias, with 398 of the 451 essential genes (88.2%) localized on chromosome I (Chr1) and 53 genes (11.8%) on chromosome II (Chr2) in *B. abortus* A19. *B. abortus* S2308 similarly displayed comparable distribution patterns, with 347 of the 393 essential genes (88.3%) residing on Chr1 and 46 genes (11.7%) residing on Chr2.

**Figure 3:**
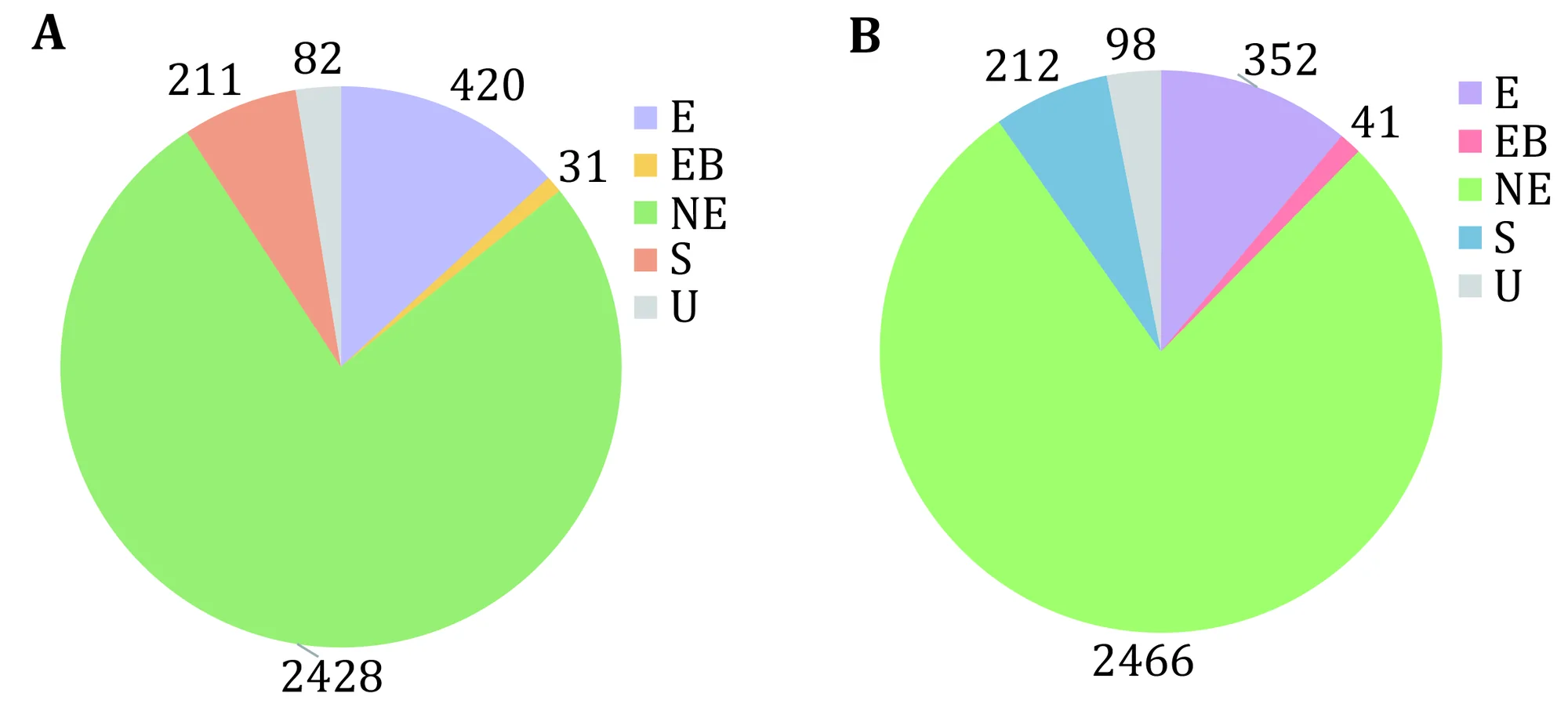
Quantitative distribution of gene functional categories in the *B. abortus* A19 (**A**) and S2308 (**B**) genomes

A detailed examination of the transposon insertion patterns within the putative essential genes revealed varying degrees of insertion tolerance. A total of 287 of the 451 (63.6%) candidate essential genes in *B. abortus* A19 contained zero transposon insertions, 150 (33.3%) harbored fewer than five insertions, and 14 (3.1%) contained more than five insertion events. Comparable patterns were observed in *B. abortus* S2308, where insertions were completely absent in 253 genes (64.4%), 131 genes (33.3%) contained fewer than five insertions, and 9 genes (2.3%) harbored more than five insertion events. The persistence of transposon insertions within certain essential genes likely reflects insertions occurring proximal to stop codons that do not disrupt critical gene function, which is consistent with the established principles of transposon mutagenesis.

### Functions of essential genes

The functional enrichment of the putative essential genes identified in *B. abortus* strains A19 and S2308 through Gumbel analysis was comprehensively analyzed using the clusterProfiler package (version 4.14.6). The results revealed that these genes are predominantly associated with five core biological processes: cofactor biosynthesis, ribosomal biogenesis, carbon metabolism, amino acid biosynthesis, and oxidative phosphorylation (Tables 1–5). The enrichment profiles of *B. abortus* A19 and S2308 were similar, indicating conserved essential gene functions between these strains (Fig. 4).

**Table 1:** Genes associated with cofactor biosynthesis identified via KEGG enrichment analysis

Gene_ID	Gene	Annotation
*B. abortus* A19		
DR186_RS02480	*fabG*	3-Oxoacyl-[acyl-carrier-protein] reductase
DR186_RS02500	*fabF*	Beta-ketoacyl-ACP synthase II
DR186_RS03970	*hemB*	Porphobilinogen synthase
DR186_RS04855	*-*	NAD kinase
DR186_RS05220	*-*	NAD+ synthase
DR186_RS05310	*folP*	Dihydropteroate synthase
DR186_RS05315	*folB*	Dihydroneopterin aldolase
DR186_RS05320	*folK*	2-Amino-4-hydroxy-6-hydroxymethyldihydropteridine diphosphokinase
DR186_RS05560	*folE*	GTP cyclohydrolase I FolE
DR186_RS05845	*lipA*	Lipoyl synthase
DR186_RS05980	*fabZ*	3-Hydroxyacyl-ACP dehydratase FabZ
DR186_RS07155	*-*	Dihydrofolate reductase
DR186_RS07600	*carA*	Glutamine-hydrolyzing carbamoyl-phosphate synthase small subunit
DR186_RS07620	*carB*	Carbamoyl-phosphate synthase large subunit
DR186_RS07930	*hemF*	Oxygen-dependent coproporphyrinogen oxidase
DR186_RS08585	*-*	Adenylosuccinate synthase
DR186_RS08925	*-*	Thiamine diphosphokinase
DR186_RS09320	*-*	Nicotinate-nucleotide adenylyltransferase
DR186_RS09495	*ubiG*	Bifunctional 2-polyprenyl-6-hydroxyphenol methylase/3-demethylubiquinol 3-O-methyltransferase UbiG
DR186_RS09555	*hemC*	Hydroxymethylbilane synthase
DR186_RS10440	*hemJ*	Protoporphyrinogen oxidase HemJ
DR186_RS10445	*hemE*	Uroporphyrinogen decarboxylase
DR186_RS10470	*coaE*	Dephospho-CoA kinase
DR186_RS10555	*coaA*	Type I pantothenate kinase
DR186_RS10905	*metK*	Methionine adenosyltransferase
DR186_RS10965	*fabB*	Beta-ketoacyl-ACP synthase I
DR186_RS10650	*-*	Folylpolyglutamate synthase/dihydrofolate synthase family protein
DR186_RS01815	*hemA*	5-Aminolevulinate synthase
DR186_RS06015	*pyrH*	UMP kinase
DR186_RS09550	*-*	Uroporphyrinogen-III synthase
DR186_RS03995	*glyA*	Serine hydroxymethyltransferase
DR186_RS05705	*coaD*	Pantetheine-phosphate adenylyltransferase
DR186_RS02275	*ubiA*	4-hydroxybenzoate octaprenyltransferase
DR186_RS04010	*-*	Riboflavin synthase
DR186_RS09610	*-*	Ubiquinone biosynthesis hydroxylase
DR186_RS04815	*-*	Cysteine desulfurase family protein
DR186_RS00585	*pncB*	Nicotinate phosphoribosyltransferase
DR186_RS03635	*ndk*	Nucleoside-diphosphate kinase
DR186_RS05895	*-*	CTP synthase
DR186_RS12010	*-*	Bifunctional riboflavin kinase/FAD synthetase
DR186_RS13325	*folD*	Bifunctional methylenetetrahydrofolate dehydrogenase/methenyltetrahydrofolate cyclohydrolase FolD
DR186_RS14195	-	Aspartate carbamoyltransferase catalytic subunit
DR186_RS14245	*lipB*	Lipoyl(octanoyl) transferase LipB
DR186_RS16105	*ubiE*	Bifunctional demethylmenaquinone methyltransferase/2-methoxy-6-polyprenyl-1%2C4-benzoquinol methylase UbiE
DR186_RS13420	*-*	Glutamate--cysteine ligase
DR186_RS11415	*hemH*	Ferrochelatase
DR186_RS16095	*coaBC*	Bifunctional phosphopantothenoylcysteine decarboxylase/phosphopantothenate--cysteine ligase CoaBC
*B. abortus* S2308		
BAB_RS16450	*pncB*	Nicotinate phosphoribosyltransferase
BAB_RS17600	*hemA*	5-Aminolevulinate synthase
BAB_RS18030	*ubiA*	4-Hydroxybenzoate octaprenyltransferase
BAB_RS18225	*fabG*	3-Oxoacyl-[acyl-carrier-protein] reductase
BAB_RS18235	*fabF*	Beta-ketoacyl-ACP synthase II
BAB_RS19675	*glyA*	Serine hydroxymethyltransferase
BAB_RS19685	*ribD*	Bifunctional diaminohydroxyphosphoribosylaminopyrimidine deaminase/5-amino-6-(5-phosphoribosylamino)uracil reductase RibD
BAB_RS19690	-	Riboflavin synthase
BAB_RS20070	*purB*	Adenylosuccinate lyase
BAB_RS20505	-	NAD kinase
BAB_RS20875	-	NAD+ synthase
BAB_RS20880	*gltX*	Glutamate--tRNA ligase
BAB_RS20950	*folP*	Dihydropteroate synthase
BAB_RS20955	*folB*	Dihydroneopterin aldolase
BAB_RS20960	*folK*	2-Amino-4-hydroxy-6-hydroxymethyldihydropteridine diphosphokinase
BAB_RS21170	*folE*	GTP cyclohydrolase I FolE
BAB_RS21285	*coaD*	Pantetheine-phosphate adenylyltransferase
BAB_RS21415	*lipA*	Lipoyl synthase
BAB_RS21545	*fabZ*	3-Hydroxyacyl-ACP dehydratase FabZ
BAB_RS21580	*pyrH*	UMP kinase
BAB_RS22685	-	Dihydrofolate reductase
BAB_RS23085	*carA*	Glutamine-hydrolyzing carbamoyl-phosphate synthase small subunit
BAB_RS23105	*carB*	Carbamoyl-phosphate synthase large subunit
BAB_RS23385	*hemF*	Oxygen-dependent coproporphyrinogen oxidase
BAB_RS24715	-	Nicotinate-nucleotide adenylyltransferase
BAB_RS24870	*ubiG*	Bifunctional 2-polyprenyl-6-hydroxyphenol methylase/3-demethylubiquinol 3-O-methyltransferase UbiG
BAB_RS24930	*-*	Uroporphyrinogen-III synthase
BAB_RS24935	*hemC*	Hydroxymethylbilane synthase
BAB_RS24990	*-*	Ubiquinone biosynthesis hydroxylase
BAB_RS25770	*hemJ*	Protoporphyrinogen oxidase HemJ
BAB_RS25775	*hemE*	Uroporphyrinogen decarboxylase
BAB_RS25880	*coaA*	Type I pantothenate kinase
BAB_RS25960	*-*	Bifunctional folylpolyglutamate synthase/dihydrofolate synthase
BAB_RS26220	*metK*	Methionine adenosyltransferase
BAB_RS26285	*fabB*	Beta-ketoacyl-ACP synthase I
BAB_RS27285	*-*	Bifunctional riboflavin kinase/FAD synthetase
BAB_RS28550	*folD*	Bifunctional methylenetetrahydrofolate dehydrogenase/methenyltetrahydrofolate cyclohydrolase FolD
BAB_RS29425	*lipB*	Lipoyl(octanoyl) transferase LipB
BAB_RS31170	*coaBC*	Bifunctional phosphopantothenoylcysteine decarboxylase/phosphopantothenate--cysteine ligase CoaBC
BAB_RS31180	*ubiE*	Bifunctional demethylmenaquinone methyltransferase/2-methoxy-6-polyprenyl-1%2C4-benzoquinol methylase UbiE

**Figure 4:**
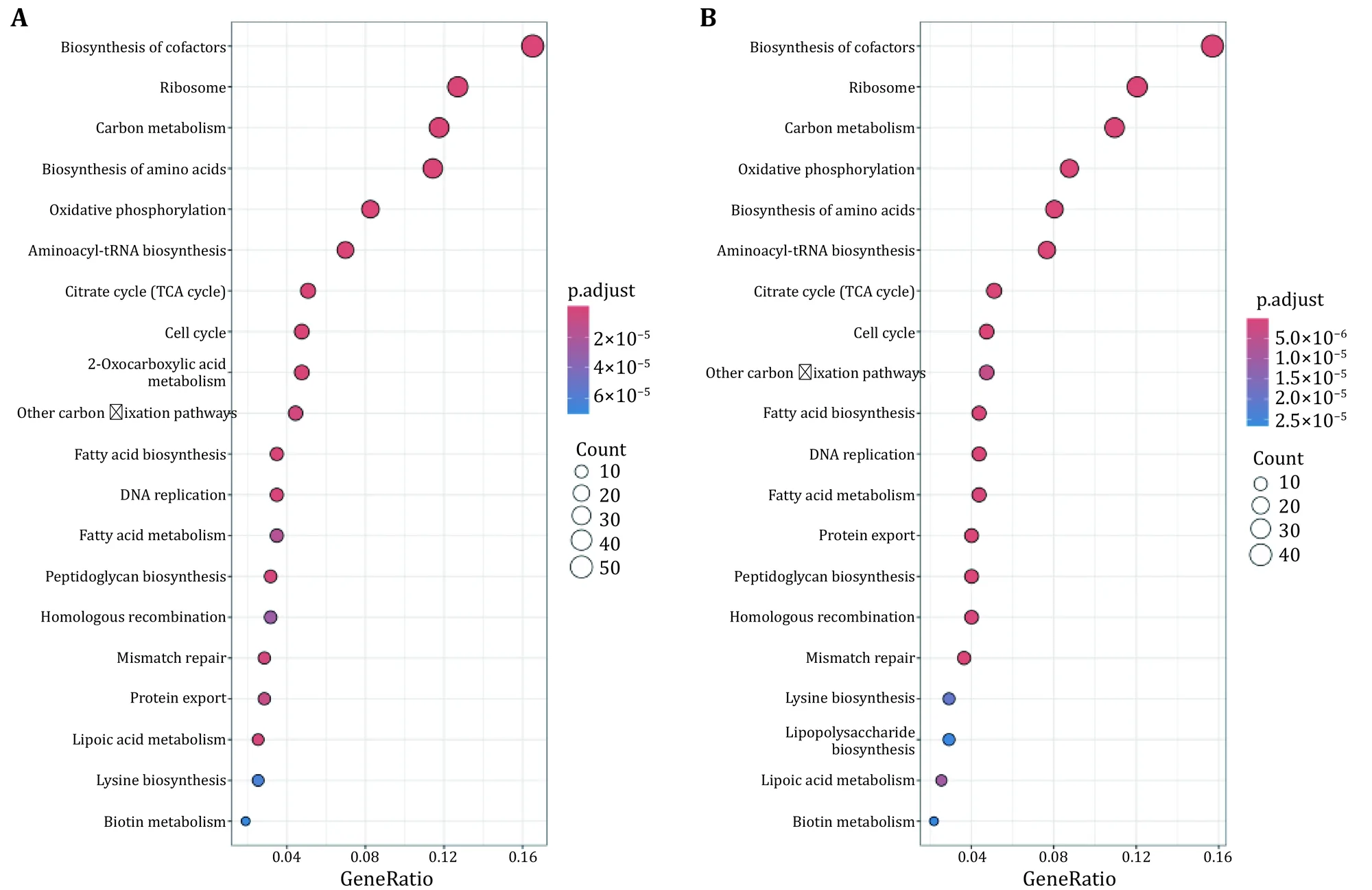
Functional categorization of putative essential genes in *B. abortus* strains A19 (**A**) and S2308 (**B**) determined through KEGG enrichment analysis

**Table 2:** Genes associated with ribosomal biogenesis identified via KEGG enrichment analysis

Gene_ID	Gene	Annotation
*B. abortus* A19		
DR186_RS02465	*rpsF*	30S ribosomal protein S6
DR186_RS04130	*rplM*	50S ribosomal protein L13
DR186_RS04320	*rpsD*	30S ribosomal protein S4
DR186_RS06025	*rpsB*	30S ribosomal protein S2
DR186_RS06260	*rpsM*	30S ribosomal protein S13
DR186_RS06285	*rpsE*	30S ribosomal protein S5
DR186_RS06290	*rplR*	50S ribosomal protein L18
DR186_RS06295	*rplF*	50S ribosomal protein L6
DR186_RS06305	*rpsN*	30S ribosomal protein S14
DR186_RS06310	*rplE*	50S ribosomal protein L5
DR186_RS06330	*rpmC*	50S ribosomal protein L29
DR186_RS06335	*rplP*	50S ribosomal protein L16
DR186_RS06340	*rpsC*	30S ribosomal protein S3
DR186_RS06345	*rplV*	50S ribosomal protein L22
DR186_RS06350	*rpsS*	30S ribosomal protein S19
DR186_RS06355	*rplB*	50S ribosomal protein L2
DR186_RS06360	*-*	50S ribosomal protein L23
DR186_RS06365	*rplD*	50S ribosomal protein L4
DR186_RS06370	*rplC*	50S ribosomal protein L3
DR186_RS06390	*rpsG*	30S ribosomal protein S7
DR186_RS06395	*rpsL*	30S ribosomal protein S12
DR186_RS06420	*rplJ*	50S ribosomal protein L10
DR186_RS06425	*rplA*	50S ribosomal protein L1
DR186_RS06430	*rplK*	50S ribosomal protein L11
DR186_RS07860	*-*	50S ribosomal protein L25/general stress protein Ctc
DR186_RS09355	*rpmA*	50S ribosomal protein L27
DR186_RS09360	*rplU*	50S ribosomal protein L21
DR186_RS10190	*rpmB*	50S ribosomal protein L28
DR186_RS10945	*rpsO*	30S ribosomal protein S15
DR186_RS06240	*rplQ*	50S ribosomal protein L17
DR186_RS06300	*rpsH*	30S ribosomal protein S8
DR186_RS00145	*rpsA*	30S ribosomal protein S1
DR186_RS10720	*rplT*	50S ribosomal protein L20
DR186_RS09660	*rplS*	50S ribosomal protein L19
DR186_RS06280	*rpmD*	50S ribosomal protein L30
DR186_RS06315	*rplX*	50S ribosomal protein L24
DR186_RS06325	*rpsQ*	30S ribosomal protein S17
DR186_RS06375	*rpsJ*	30S ribosomal protein S10
DR186_RS09010	*rpmF*	50S ribosomal protein L32
DR186_RS09225	*rpsP*	30S ribosomal protein S16
*B. abortus* S2308		
BAB_RS16055	*rpsA*	30S ribosomal protein S1
BAB_RS19990	*rpsD*	30S ribosomal protein S4
BAB_RS21590	*rpsB*	30S ribosomal protein S2
BAB_RS21795	*rplQ*	50S ribosomal protein L17
BAB_RS21805	*rpsK*	30S ribosomal protein S11
BAB_RS21810	*rpsM*	30S ribosomal protein S13
BAB_RS21830	*rpmD*	50S ribosomal protein L30
BAB_RS21835	*rpsE*	30S ribosomal protein S5
BAB_RS21840	*rplR*	50S ribosomal protein L18
BAB_RS21845	*rplF*	50S ribosomal protein L6
BAB_RS21850	*rpsH*	30S ribosomal protein S8
BAB_RS21855	*rpsN*	30S ribosomal protein S14
BAB_RS21860	*rplE*	50S ribosomal protein L5
BAB_RS21865	*rplX*	50S ribosomal protein L24
BAB_RS21870	*rplN*	50S ribosomal protein L14
BAB_RS21875	*rpsQ*	30S ribosomal protein S17
BAB_RS21885	*rplP*	50S ribosomal protein L16
BAB_RS21890	*rpsC*	30S ribosomal protein S3
BAB_RS21895	*rplV*	50S ribosomal protein L22
BAB_RS21900	*rpsS*	30S ribosomal protein S19
BAB_RS21905	*rplB*	50S ribosomal protein L2
BAB_RS21910	*-*	50S ribosomal protein L23
BAB_RS21920	*rplC*	50S ribosomal protein L3
BAB_RS21940	*rpsG*	30S ribosomal protein S7
BAB_RS21945	*rpsL*	30S ribosomal protein S12
BAB_RS21970	*rplJ*	50S ribosomal protein L10
BAB_RS21975	*rplA*	50S ribosomal protein L1
BAB_RS21980	*rplK*	50S ribosomal protein L11
BAB_RS23315	*-*	50S ribosomal protein L25/general stress protein Ctc
BAB_RS24420	*rpmF*	50S ribosomal protein L32
BAB_RS24630	*rpsP*	30S ribosomal protein S16
BAB_RS25030	*rplS*	50S ribosomal protein L19
BAB_RS25530	*rpmB*	50S ribosomal protein L28

**Table 3:** Genes associated with carbon metabolism identified via KEGG enrichment analysis

Gene_ID	Gene	Annotation
*B. abortus* A19		
DR186_RS01655	*pgi*	Glucose-6-phosphate isomerase
DR186_RS04415	*rpe*	Ribulose-phosphate 3-epimerase
DR186_RS04705	*accC*	Acetyl-CoA carboxylase biotin carboxylase subunit
DR186_RS04710	*accB*	Acetyl-CoA carboxylase biotin carboxyl carrier protein
DR186_RS05195	*rpiA*	Ribose-5-phosphate isomerase RpiA
DR186_RS05870	*pdhA*	Pyruvate dehydrogenase (acetyl-transferring) E1 component subunit alpha
DR186_RS05960	*gltA*	Citrate synthase
DR186_RS07125	*serB*	Phosphoserine phosphatase SerB
DR186_RS07845	*-*	Ribose-phosphate pyrophosphokinase
DR186_RS08790	*gap*	Type I glyceraldehyde-3-phosphate dehydrogenase
DR186_RS09140	*fsa*	Fructose-6-phosphate aldolase
DR186_RS09630	*sdhA*	Succinate dehydrogenase flavoprotein subunit
DR186_RS09735	*odhB*	2-Oxoglutarate dehydrogenase complex dihydrolipoyllysine-residue succinyltransferase
DR186_RS09745	*sucD*	Succinate--CoA ligase subunit alpha
DR186_RS09750	*sucC*	ADP-forming succinate--CoA ligase subunit beta
DR186_RS09755	*mdh*	Malate dehydrogenase
DR186_RS10655	*accD*	Acetyl-CoA carboxylase%2C carboxyltransferase subunit beta
DR186_RS08785	*tkt*	Transketolase
DR186_RS09740	*-*	2-Oxoglutarate dehydrogenase E1 component
DR186_RS06200	*-*	NADP-dependent isocitrate dehydrogenase
DR186_RS05860	*-*	Pyruvate dehydrogenase complex dihydrolipoamide acetyltransferase
DR186_RS09625	*-*	Succinate dehydrogenase iron-sulfur subunit
DR186_RS10275	*-*	Acetyl-CoA carboxylase carboxyltransferase subunit alpha
DR186_RS00485	*acnA*	Aconitate hydratase AcnA
DR186_RS05865	*-*	Pyruvate dehydrogenase complex E1 component subunit beta
DR186_RS05885	*eno*	Phosphopyruvate hydratase
DR186_RS03995	*glyA*	Serine hydroxymethyltransferase
DR186_RS09635	*sdhD*	Succinate dehydrogenase%2C hydrophobic membrane anchor protein
DR186_RS06505	*cysE*	Serine O-acetyltransferase
DR186_RS09640	*sdhC*	Succinate dehydrogenase%2C cytochrome b556 subunit
DR186_RS05445	*-*	Cysteine synthase A
DR186_RS13325	*folD*	Bifunctional methylenetetrahydrofolate dehydrogenase/methenyltetrahydrofolate cyclohydrolase FolD
DR186_RS13335	*pgl*	6-Phosphogluconolactonase
DR186_RS13340	*zwf*	Glucose-6-phosphate dehydrogenase
DR186_RS11575	*gndA*	NADP-dependent phosphogluconate dehydrogenase
*B. abortus* S2308		
BAB_RS16360	*acnA*	Aconitate hydratase AcnA
BAB_RS17440	*pgi*	Glucose-6-phosphate isomerase
BAB_RS19675	*glyA*	Serine hydroxymethyltransferase
BAB_RS20075	*rpe*	Ribulose-phosphate 3-epimerase
BAB_RS20350	*accC*	Acetyl-CoA carboxylase biotin carboxylase subunit
BAB_RS20355	*accB*	Acetyl-CoA carboxylase biotin carboxyl carrier protein
BAB_RS20890	*-*	NADP-dependent malic enzyme
BAB_RS21430	*-*	Pyruvate dehydrogenase complex dihydrolipoamide acetyltransferase
BAB_RS21435	*-*	Pyruvate dehydrogenase complex E1 component subunit beta
BAB_RS21440	*pdhA*	Pyruvate dehydrogenase (acetyl-transferring) E1 component subunit alpha
BAB_RS21455	*eno*	Phosphopyruvate hydratase
BAB_RS21525	*gltA*	Citrate synthase
BAB_RS21755	*-*	NADP-dependent isocitrate dehydrogenase
BAB_RS22655	*serB*	Phosphoserine phosphatase SerB
BAB_RS23305	*-*	Ribose-phosphate pyrophosphokinase
BAB_RS24225	*tkt*	Transketolase
BAB_RS24230	*gap*	Type I glyceraldehyde-3-phosphate dehydrogenase
BAB_RS25000	*-*	Succinate dehydrogenase iron-sulfur subunit
BAB_RS25005	*sdhA*	Succinate dehydrogenase flavoprotein subunit
BAB_RS25015	*sdhC*	Succinate dehydrogenase%2C cytochrome b556 subunit
BAB_RS25105	*odhB*	2-Oxoglutarate dehydrogenase complex dihydrolipoyllysine-residue succinyltransferase
BAB_RS25110	*-*	2-Oxoglutarate dehydrogenase E1 component
BAB_RS25115	*sucD*	Succinate--CoA ligase subunit alpha
BAB_RS25120	*sucC*	ADP-forming succinate--CoA ligase subunit beta
BAB_RS25125	*mdh*	Malate dehydrogenase
BAB_RS25605	*-*	Acetyl-CoA carboxylase carboxyltransferase subunit alpha
BAB_RS25965	*accD*	Acetyl-CoA carboxylase%2C carboxyltransferase subunit beta
BAB_RS26870	*gndA*	NADP-dependent phosphogluconate dehydrogenase
BAB_RS28550	*folD*	Bifunctional methylenetetrahydrofolate dehydrogenase/methenyltetrahydrofolate cyclohydrolase FolD
BAB_RS28565	*zwf*	Glucose-6-phosphate dehydrogenase

**Table 4:** Genes associated with amino acid biosynthesis identified via KEGG enrichment analysis

Gene_ID	Gene	Annotation
*B. abortus* A19		
DR186_RS00130	*aroA*	3-Phosphoshikimate 1-carboxyvinyltransferase
DR186_RS01735	*argF*	Ornithine carbamoyltransferase
DR186_RS02625	*thrC*	Threonine synthase
DR186_RS03405	*dapA*	4-Hydroxy-tetrahydrodipicolinate synthase
DR186_RS04415	*rpe*	Ribulose-phosphate 3-epimerase
DR186_RS04715	*aroQ*	Type II 3-dehydroquinate dehydratase
DR186_RS05195	*rpiA*	Ribose-5-phosphate isomerase RpiA
DR186_RS05960	*gltA*	Citrate synthase
DR186_RS07125	*serB*	Phosphoserine phosphatase SerB
DR186_RS07650	*-*	Pyridoxal phosphate-dependent aminotransferase
DR186_RS07845	*-*	Ribose-phosphate pyrophosphokinase
DR186_RS08790	*gap*	Type I glyceraldehyde-3-phosphate dehydrogenase
DR186_RS09140	*fsa*	Fructose-6-phosphate aldolase
DR186_RS09230	*-*	Chorismate mutase
DR186_RS09325	*-*	Glutamate-5-semialdehyde dehydrogenase
DR186_RS09490	*-*	Aspartate kinase
DR186_RS09780	*dapF*	Diaminopimelate epimerase
DR186_RS10905	*metK*	Methionine adenosyltransferase
DR186_RS08785	*tkt*	Transketolase
DR186_RS06200	*-*	NADP-dependent isocitrate dehydrogenase
DR186_RS07110	*-*	Acetolactate synthase 3 large subunit
DR186_RS04115	*argC*	N-acetyl-gamma-glutamyl-phosphate reductase
DR186_RS05170	*glnA*	Type I glutamate--ammonia ligase
DR186_RS00485	*acnA*	Aconitate hydratase AcnA
DR186_RS02330	*aroC*	Chorismate synthase
DR186_RS05885	*eno*	Phosphopyruvate hydratase
DR186_RS03995	*glyA*	Serine hydroxymethyltransferase
DR186_RS06505	*cysE*	Serine O-acetyltransferase
DR186_RS10255	*aroB*	3-Dehydroquinate synthase
DR186_RS01730	*-*	Aspartate aminotransferase family protein
DR186_RS05210	*-*	Class II 3-deoxy-7-phosphoheptulonate synthase
DR186_RS05445	*-*	Cysteine synthase A
DR186_RS10020	*argH*	Argininosuccinate lyase
DR186_RS15915	*dapD*	2%2C3%2C4%2C5-tetrahydropyridine-2%2C6-dicarboxylate N-succinyltransferase
DR186_RS15900	*argB*	Acetylglutamate kinase
DR186_RS15925	*dapE*	Succinyl-diaminopimelate desuccinylase
*B. abortus* S2308		
BAB_RS16045	*aroA*	3-Phosphoshikimate 1-carboxyvinyltransferase
BAB_RS16360	*acnA*	Aconitate hydratase AcnA
BAB_RS17510	*-*	Aspartate aminotransferase family protein
BAB_RS18350	*thrC*	Threonine synthase
BAB_RS19125	*dapA*	4-Hydroxy-tetrahydrodipicolinate synthase
BAB_RS19675	*glyA*	Serine hydroxymethyltransferase
BAB_RS20075	*rpe*	Ribulose-phosphate 3-epimerase
BAB_RS20360	*aroQ*	Type II 3-dehydroquinate dehydratase
BAB_RS21455	*eno*	Phosphopyruvate hydratase
BAB_RS21525	*gltA*	Citrate synthase
BAB_RS21755	*-*	NADP-dependent isocitrate dehydrogenase
BAB_RS22655	*serB*	Phosphoserine phosphatase SerB
BAB_RS23305	*-*	Ribose-phosphate pyrophosphokinase
BAB_RS24225	*tkt*	Transketolase
BAB_RS24230	*gap*	Type I glyceraldehyde-3-phosphate dehydrogenase
BAB_RS24635	*-*	Chorismate mutase
BAB_RS24720	*-*	Glutamate-5-semialdehyde dehydrogenase
BAB_RS24865	*-*	Aspartate kinase
BAB_RS25140	*dapF*	Diaminopimelate epimerase
BAB_RS26220	*metK*	Methionine adenosyltransferase
BAB_RS28035	*-*	Aspartate-semialdehyde dehydrogenase
BAB_RS31000	*dapD*	2%2C3%2C4%2C5-tetrahydropyridine-2%2C6-dicarboxylate N-succinyltransferase

**Table 5:** Genes associated with oxidative phosphorylation identified via KEGG enrichment analysis

Gene_ID	Gene	Annotation
*B. abortus* A19		
DR186_RS02120	-	F0F1 ATP synthase subunit A
DR186_RS02130	-	F0F1 ATP synthase subunit B
DR186_RS04195	-	NADH-quinone oxidoreductase subunit A
DR186_RS04200	-	NADH-quinone oxidoreductase subunit B family protein
DR186_RS04205	-	NADH-quinone oxidoreductase subunit C
DR186_RS04210	-	NADH-quinone oxidoreductase subunit D
DR186_RS04215	*nuoE*	NADH-quinone oxidoreductase subunit NuoE
DR186_RS04220	*nuoF*	NADH-quinone oxidoreductase subunit NuoF
DR186_RS04225	*nuoG*	NADH-quinone oxidoreductase subunit NuoG
DR186_RS04230	*nuoH*	NADH-quinone oxidoreductase subunit NuoH
DR186_RS04235	*nuoI*	NADH-quinone oxidoreductase subunit NuoI
DR186_RS04240	*-*	NADH-quinone oxidoreductase subunit J
DR186_RS04245	*nuoK*	NADH-quinone oxidoreductase subunit NuoK
DR186_RS04250	*nuoL*	NADH-quinone oxidoreductase subunit L
DR186_RS04260	*nuoN*	NADH-quinone oxidoreductase subunit NuoN
DR186_RS09115	*-*	F0F1 ATP synthase subunit gamma
DR186_RS09630	*sdhA*	Succinate dehydrogenase flavoprotein subunit
DR186_RS09120	*atpA*	F0F1 ATP synthase subunit alpha
DR186_RS09625	*-*	Succinate dehydrogenase iron-sulfur subunit
DR186_RS09110	*atpD*	F0F1 ATP synthase subunit beta
DR186_RS10090	*ppa*	Inorganic diphosphatase
DR186_RS09635	*sdhD*	Succinate dehydrogenase%2C hydrophobic membrane anchor protein
DR186_RS09640	*sdhC*	Succinate dehydrogenase%2C cytochrome b556 subunit
DR186_RS02125	*-*	F0F1 ATP synthase subunit C
DR186_RS04255	*-*	NADH-quinone oxidoreductase subunit M
DR186_RS09125	*-*	F0F1 ATP synthase subunit delta
*B. abortus* S2308		
BAB_RS17890	*-*	F0F1 ATP synthase subunit A
BAB_RS17895	*-*	F0F1 ATP synthase subunit C
BAB_RS17900	*-*	F0F1 ATP synthase subunit B
BAB_RS19860	*-*	NuoB/complex I 20 kDa subunit family protein
BAB_RS19865	*-*	NADH-quinone oxidoreductase subunit C
BAB_RS19870	*-*	NADH-quinone oxidoreductase subunit D
BAB_RS19875	*nuoE*	NADH-quinone oxidoreductase subunit NuoE
BAB_RS19880	*nuoF*	NADH-quinone oxidoreductase subunit NuoF
BAB_RS19885	*nuoG*	NADH-quinone oxidoreductase subunit NuoG
BAB_RS19890	*nuoH*	NADH-quinone oxidoreductase subunit NuoH
BAB_RS19895	*nuoI*	NADH-quinone oxidoreductase subunit NuoI
BAB_RS19900	*-*	NADH-quinone oxidoreductase subunit J
BAB_RS19905	*nuoK*	NADH-quinone oxidoreductase subunit NuoK
BAB_RS19910	*nuoL*	NADH-quinone oxidoreductase subunit L
BAB_RS19915	*-*	NADH-quinone oxidoreductase subunit M
BAB_RS19920	*nuoN*	NADH-quinone oxidoreductase subunit NuoN
BAB_RS24515	*atpD*	F0F1 ATP synthase subunit beta
BAB_RS24520	*-*	F0F1 ATP synthase subunit gamma
BAB_RS24525	*atpA*	F0F1 ATP synthase subunit alpha
BAB_RS24530	*-*	F0F1 ATP synthase subunit delta
BAB_RS25000	*-*	Succinate dehydrogenase iron-sulfur subunit
BAB_RS25005	*sdhA*	Succinate dehydrogenase flavoprotein subunit
BAB_RS25015	*sdhC*	Succinate dehydrogenase%2C cytochrome b556 subunit
BAB_RS25435	*ppa*	Inorganic diphosphatase

### Cofactor biosynthesis and metabolic enzyme functions

Cofactor biosynthesis was the most enriched functional category, reflecting its fundamental role in bacterial viability. Cofactors are essential structural components that bind inactive apoptoenzymes to form catalytically active holoenzymes, enabling critical physiological processes. These molecules directly regulate bacterial proliferation through modulating enzymatic activity, metabolic flux, and redox homeostasis (Sun *et al*. 2023). Cofactors can be functionally categorized into three distinct types based on their chemical structures and catalytic roles: catalysts, carriers, and substrates (Wang *et al*. 2019).

Dephospho-coenzyme A (CoA) kinase is an example of catalytic cofactor biosynthesis among the essential genes identified. Dephospho-CoA kinase enzyme catalyzes the ATP-dependent phosphorylation of dephospho-CoA to generate CoA, which represents the final step in CoA biosynthesis. This biosynthetic enzyme is indispensable for bacterial growth, given the extensive participation of CoA in diverse metabolic pathways, being highly conserved across prokaryotic and eukaryotic organisms (Shimosaka *et al*. 2019).

The essential gene library revealed numerous additional enzymes involved in cofactor metabolism and energy transduction, such as NAD+ kinase, NAD+ synthase, dihydropteroate synthase, 5-aminolevulinate synthase, nicotinate phosphoribosyltransferase, and serine hydroxymethyltransferase. These enzymes have various implications for bioengineering applications and antimicrobial target development. The overexpression of NAD+ synthase and nicotinate phosphoribosyltransferase in industrial strains in the bioengineering context enhances intracellular NAD(H) and NADP(H) concentrations, increasing the productivity of bacterial transformation systems (Jaroensuk *et al*. 2024). Dihydropteroate synthase is the primary molecular target of sulfonamide antibiotics. Dihydropteroate synthase catalyzes the condensation of p-aminobenzoic acid and dihydropteridine pyrophosphate to form dihydropteroate, the immediate precursor in tetrahydrofolate synthesis (Bermingham and Derrick 2002). Sulfonamides exert antimicrobial effects through competitively inhibiting dihydropteroate synthase, effectively disrupting folate biosynthesis in pathogenic bacteria (Duysak *et al*. 2023). However, the specific function of dihydropteroate synthase in *B. abortus* has not been validated.

### Core cellular processes and carbon metabolism

Ribosomal biogenesis represents another major functional category that was enriched among the essential genes, reflecting the fundamental need to synthesize protein for bacterial viability. The identified essential proteins were exclusively involved in the assembly and maturation of 30S and 50S ribosomal subunits, underscoring the critical necessity of the translational machinery for cellular function.

Carbon metabolic pathways, including glycolysis, the tricarboxylic acid cycle, and the pentose phosphate pathway, provide the metabolic foundation required for bacterial energy production and biosynthetic precursor generation. The key enzymes identified in these interconnected pathways included transketolase from the pentose phosphate pathway; glucose-6-phosphate isomerase and triosephosphate isomerase from glycolysis; and citrate synthase, malate dehydrogenase, and NADP+-dependent isocitrate dehydrogenase from the tricarboxylic acid cycle. Additionally, essential genes involved in purine nucleotide biosynthesis, including ribose-phosphate pyrophosphokinase, and various enzymes involved in one-carbon metabolism were identified as critical for maintaining cellular nucleotide pools.

### Amino acid biosynthesis and oxidative phosphorylation

The essential gene repertoire comprises numerous enzymes that are indispensable for amino acid biosynthesis, highlighting the need for *de novo* amino acid production under standard growth conditions. An example is chorismate mutase, a pivotal enzyme involved in aromatic amino acid biosynthesis that catalyzes the Claisen rearrangement of chorismate to prephenate, the immediate precursor of phenylalanine and tyrosine synthesis (van Neer *et al*. 2025). This enzyme participates in the shikimate pathway and is a potential target for the development of antibacterial inhibitors against pathogenic mycobacteria (Russ *et al*. 2020).

Most of the identified essential genes within the oxidative phosphorylation pathway encode the structural and catalytic subunits of the NADH dehydrogenase complex and ATP synthase. This reflects the fundamental need for aerobic energy production in *Brucella* species. These findings emphasize the critical dependence of *B. abortus* on oxidative metabolism for maintaining cellular energy homeostasis.

These five functional categories collectively encompassed most of the enrichment signals observed in the analysis. The representation of the other metabolic and biosynthetic pathways was substantially lower than that of the essential gene sets. This distribution pattern indicates that *B. abortus* essentiality is primarily driven by the core metabolic processes, protein synthesis machinery, and fundamental biosynthetic capabilities required for bacterial survival under laboratory culture conditions.

## DISCUSSION

Tn-seq technology has been applied for screening virulence genes, growth factors, and drug resistance targets. We constructed a whole-genome mutant library for *B. abortus* strains A19 and S2308. The insertion site saturation exceeded 60% in both mutant libraries, indicating the high quality of the mutant library and the reliability of the subsequent results. The incubation time on the plates during library construction was strictly limited to 72 h to prevent sufficient growth of insertion mutants with growth defects at detrimental sites.

Although ribosomal protein genes are evolutionarily conserved, insertions were observed in rplQ, rpsH, rplT, and rplS within the *B. abortus* A19 library, which probably did not abolish normal function. Insertions were similarly observed in rpsA, rplS, and rplQ in the *B. abortus* S2308 library. A study on a *B. melitensis* NI mutant library also documented transposon insertions in the ribosomal protein genes *rpsB* and *rpsI* without affecting gene function, suggesting that certain ribosomal protein genes possess a degree of tolerance to transposon insertion (Cloeckaert *et al*. 2002; De *et al*. 2017).

Several of the potentially essential genes among those identified in the two libraries represented important drug targets, whereas others were associated with drug resistance or virulence in other pathogens. For example, dihydropteroate synthase is a key target for sulfonamide drugs (Venkatesan *et al*. 2023). MurC is a potential target for curcumin derivatives in *Salmonella typhi* (Debroy and Ramaiah 2022). FabF is a target for acyl sulfonamides in chlamydia (Mojica *et al*. 2017).

Several genes involved in lipopolysaccharide biosynthesis were identified in the two libraries. For instance, LptE in *Pseudomonas aeruginosa* promotes LptD maturation and stability (Lo Sciuto *et al*. 2018). LptE depletion not only reduces the virulence of *P. aeruginosa* but also causes severe membrane permeability defects, leading to a marked increase in susceptibility to Rifampin. Moreover, the susceptibility to Tetracycline and β-lactam antibiotics increased after LptE depletion (Shen *et al*. 2019). LptE is thus an essential gene in *P. aeruginosa* (Bolla *et al*. 2011). Lipopolysaccharide is a crucial antigen in *Brucella* that plays a key role in its pathogenicity. LptE may play a similar role in *Brucella* based on sequence homology analysis (Conde-Álvarez *et al*. 2013).

We constructed random mutant libraries of the *B. abortus* strains A19 and S2308. We used Tn-seq analysis to identify 451 and 393 putative essential genes required for survival on TSA plates in *B. abortus* strains A19 and S2308, respectively. Under different stress conditions or during host-pathogen interactions, the essentiality of certain genes may change, transitioning from non-essential to essential. These resources provide a foundation for further studying the essential genes governing *Brucella* survival *in vitro* and *in vivo*, as well as for developing novel vaccine candidates and drug targets against brucellosis.

## MATERIALS AND METHODS

### Bacterial strains, culture conditions, and plasmids

*B. abortus* strains A19 and S2308 were obtained from cryogenic laboratory storage and maintained in tryptic soy broth (TSB) or tryptic soy agar (Difco, BD, Franklin Lakes, NJ, USA) at 37°C under orbital shaking at 180 r/min. Transformants were selected using kanamycin sulfate (50 μg/mL). *Escherichia coli* strains were cultivated in Luria-Bertani medium supplemented with strain-specific antibiotics. Auxotrophic strain WM3064 required additional supplementation with 2,6-diaminopimelic acid (50 μg/mL; Sigma-Aldrich). Kanamycin (25 μg/mL) was used for selection. All bacterial cultures were preserved at −80°C in culture medium containing 35% (*v*/*v*) glycerol.

### Constructing *B. abortus* A19 and *S2308* mutant library

Comprehensive transposon insertion libraries were separately constructed for *B. abortus* strains A19 and S2308 using the *Mariner himar1C9* transposase system delivered via the suicide vector pSam_Cam. Conjugative transfer was performed using *E. coli* WM3064 as the donor organism.

The donor strain harboring pSam_Mariner was cultured for 8 h in Luria-Bertani medium supplemented with kanamycin (25 μg/mL) and 2,6-diaminopimelic acid (50 μg/mL). The recipient *B. abortus* strain (A19 or S2308) was independently cultured in TSB medium for 48 h at 37°C to construct each library. Both bacterial cultures were harvested via centrifugation and washed with TSB. The resulting cell suspensions were collected into separate 50 mL centrifuge tubes.

Donor *E. coli* cells were separately mixed for conjugative mating with recipient *B. abortus* A19 or S2308 cells in a 3:1 volume ratio in 1.5 mL microcentrifuge tubes and transferred onto TSA plates, which were overlaid with nitrocellulose membranes. The mating plates were incubated at 37°C for 12 h. The bacteria were washed from the nitrocellulose membranes following conjugation using TSB, adjusted to a final volume of 20 mL, and induced with isopropyl β-D-1-thiogalactopyranoside at a final concentration of 1 mmol/L from a 1 mol/L stock solution under shaking conditions (180 r/min, 37°C) for 3 h.

Postinduction cells were pelleted via centrifugation (5000 r/min, 5 min), resuspended in fresh TSB to terminate isopropyl β-D-1-thiogalactopyranoside activity, and plated onto TSA containing kanamycin sulfate (50 μg/mL) using sterile glass beads to ensure uniform distribution. The transposon-positive colonies from each strain after 72 h of incubation at 37°C were separately processed to preserve the libraries. All colonies were harvested into 50 mL centrifuge tubes using TSB. A 15-mL aliquot was heat-inactivated (90°C, 20 min) for each library for the subsequent genomic sequencing analysis (Jiang *et al*. 2025). The remaining cell suspension was mixed with an equal volume of sterile 50% (*v*/*v*) glycerol, aliquoted into 2 mL cryovials, and archived at −80°C for long-term storage (Fig. 5).

**Figure 5:**
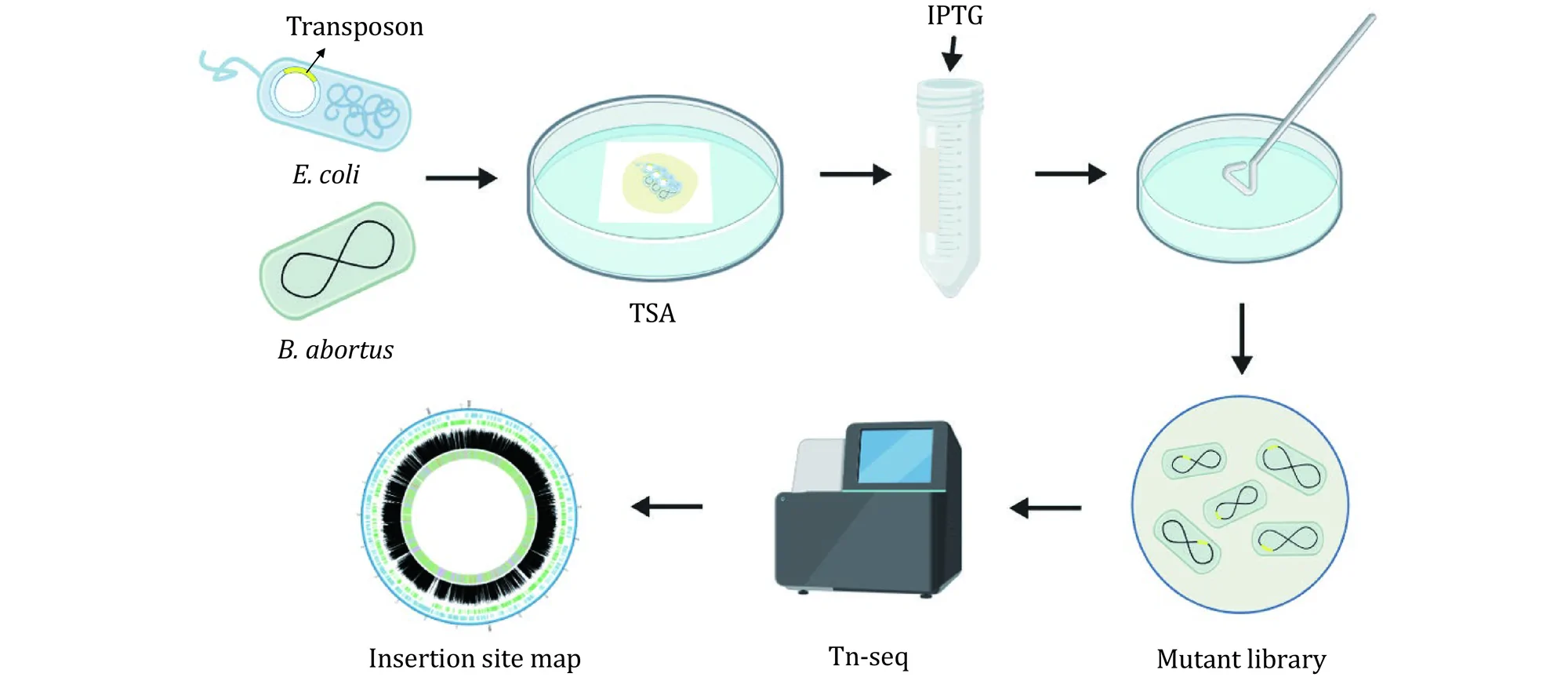
Schematic diagram of mutant library construction

### Mutant library screening and characterization

The genomic DNA was extracted from the inactivated samples using a TIANamp Bacterial DNA Kit, and its quality was assessed. The DNA was randomly fragmented into 200–500 bp segments, which was followed by end repair and A-tailing. Adaptors were ligated to the repaired ends, and target fragments were enriched through primary PCR amplification using the transposon-specific primer R1 and adapter universal primer Tn-F. The amplified products were purified using 1X magnetic beads. Secondary PCR amplification was then performed using the P5 universal primer and the P7 index primer, followed by another 1X magnetic bead purification to obtain the final sequencing library. The quality of the resulting library was verified. The libraries were pooled after quality control according to the effective concentration and sequencing depth requirements for Illumina PE150 sequencing (Hangzhou Watson Biotechnologies). Sequence reads were filtered using the TPP tool within the TRANSIT software package based on primer-specific criteria to retain qualified sequences (Shen *et al*. 2016). The filtered reads were aligned against the reference genome sequence to map the precise insertion sites. The gene annotation files were converted to a TRANSIT-compatible format. Gene essentiality was determined using Gumbel analysis in TRANSIT, yielding separate essential gene calls based on the Gumbel (E) and binomial (EB) statistical models (DeJesus *et al*. 2015).

### Putative essential gene enrichment analysis

After the transit Gumbel analysis, genes classified as “E” or “EB” were considered essential genes in the sample. KEGG enrichment analysis was performed on the identified essential genes using the clusterProfiler package (v4.14.6). The results from the enrichment analysis were visualized using the “dotplot” function.
